# Semitransparent inverted polymer solar cells employing a sol-gel-derived TiO_2_ electron-selective layer on FTO and MoO_3_/Ag/MoO_3_ transparent electrode

**DOI:** 10.1186/1556-276X-9-579

**Published:** 2014-10-17

**Authors:** Fumin Li, Chong Chen, Furui Tan, Chunxi Li, Gentian Yue, Liang Shen, Weifeng Zhang

**Affiliations:** 1 Key Laboratory of Photovoltaic Materials, Department of Physics and Electronics, Henan University, Kaifeng 475004, People's Republic of China; 2 State Key Laboratory on Integrated Optoelectronics, College of Electronic Science and Engineering, Jilin University, 2699 Qianjin Street, Changchun 130012, People's Republic of China

**Keywords:** Polymer solar cell, Indium tin oxide, Nanocrystalline titanium oxide, Power conversion efficiency

## Abstract

We report a new semitransparent inverted polymer solar cell (PSC) with a structure of glass/FTO/nc-TiO_2_/P3HT:PCBM/MoO_3_/Ag/MoO_3_. Because high-temperature annealing which decreased the conductivity of indium tin oxide (ITO) must be handled in the process of preparation of nanocrystalline titanium oxide (nc-TiO_2_), we replace glass/ITO with a glass/fluorine-doped tin oxide (FTO) substrate to improve the device performance. The experimental results show that the replacing FTO substrate enhances light transmittance between 400 and 600 nm and does not change sheet resistance after annealing treatment. The dependence of device performances on resistivity, light transmittance, and thickness of the MoO_3_/Ag/MoO_3_ film was investigated. High power conversion efficiency (PCE) was achieved for FTO substrate inverted PSCs, which showed about 75% increase compared to our previously reported ITO substrate device at different thicknesses of the MoO_3_/Ag/MoO_3_ transparent electrode films illuminated from the FTO side (bottom side) and about 150% increase illuminated from the MoO_3_/Ag/MoO_3_ side (top side).

## Background

Bulk heterojunction (BHJ) polymer solar cells (PSCs) have been extensively investigated as a new energy substitute due to their low cost, solution processing capability, and flexibility in fabricating large-area devices [1-6]. So far, the power conversion efficiency (PCE) of BHJ PSCs has recently achieved 9.2% or more [7,8]. It is very close to the commercialization level. However, there are some factors limiting the efficiency of PSCs, such as low absorption efficiency and narrow absorption range, short exciton diffusion length, low charge carrier mobility, and so on [9,10]. One of the possible strategies to increase its PCE is to stack two or more cells with different spectra response together as tandem solar cells [8,11]. It is particularly important to study semitransparent solar cells on the investigation of tandem solar cells. Meanwhile, semitransparent BHJ PSCs are also interesting for other applications, such as power-generating windows [12].

The semitransparent BHJ PSCs require transparent electrodes on both bottom and top sides. There are many researches that focus on top transparent electrodes while the bottom ones typically use indium tin oxide (ITO) electrodes with high transparency in the visible light region [13-18]. And these top transparent electrodes may use one type of thin metal (such as the highly reflective anode Al (100 nm) replaced by a transparent layer of Ag (20 nm) or by a transparent layer of Au (12 nm) [13,14]), may stack two or more thin metals (such as Al/Au (0.5 nm/15 nm) [15,16]), or may use multilayer composite structure (such as PEDOT:PSS/PH1000/WO_*x*_ (40 nm/70 nm/20 nm), WO_3_/Ag/WO_3_ (10 nm/13 nm/40 nm) [17,18]) and so on.

In our previous reports [19], ITO substrate semitransparent inverted PSCs were studied. The conductivity of ITO decreased because high-temperature (500°C) annealing must be handled during the preparation of nanocrystalline titanium oxide (nc-TiO_2_). This report focuses on the fluorine-doped tin oxide (FTO) substrate semitransparent inverted PSCs employing a sol-gel-derived TiO_2_ electron-selective layer (ESL) and with a multilayer anode structure of MoO_3_/Ag/MoO_3_. The inner MoO_3_ layer, which served as a hole transport material (HTM), is inserted between the active layer and Ag to enhance hole collection, and the outer MoO_3_ layer is used as a top capping layer to enhance light coupling.

The results show that the replacing FTO substrate enhances light transmittance between 400 and 600 nm but does not change sheet resistance after annealing treatment. Compared to our reported ITO substrate inverted PSCs, high PCE about 75% increase was achieved for the FTO substrate device when illuminated from the FTO side (bottom side) and about 150% increase done when illuminated from the MoO_3_/Ag/MoO_3_ side (top side).

## Methods

**Figure 1:**
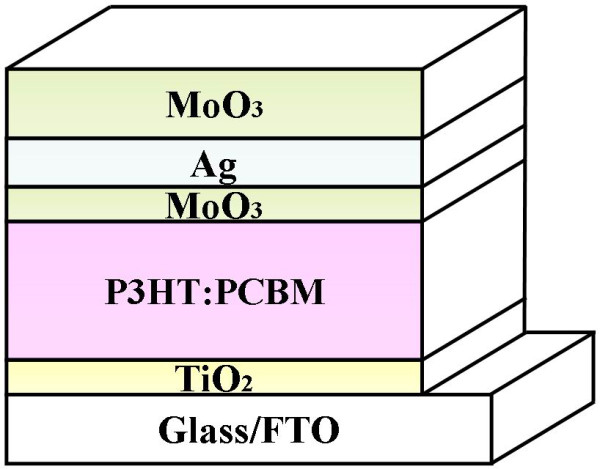
Schematic structure drawing of semitransparent inverted polymer solar cells.

The photovoltaic device has a structure of FTO/nc-TiO_2_/P3HT:PCBM/MoO_3_/Ag/MoO_3_, (P3HT, Luminescence Technology Co., Palo Alto, CA, USA; 95 + % regioregular, electronic grade, PCBM, Luminescence Technology Co., Palo Alto, CA, USA, 99.5 + %) as shown schematically in Figure 1. The FTO conducting glass substrate (with a sheet resistance of <15 Ω/□) was pre-cleaned using acetone, ethanol, and deionized (DI) water for 15 min each. Anatase phase TiO_2_ thin films were prepared through a sol-gel method similar to our previous papers [20,21]. The procedure for the preparation of TiO_2_-sol involved the dissolution of 10 ml Ti(OC_4_H_9_)_4_ in 60 ml ethanol (C_2_H_5_OH), followed by adding 10 ml acetyl acetone. Then, a solution, composed of 30 ml C_2_H_5_OH, 2 ml DI water, and 2 ml hydrochloric acid (HCl) with a density of 0.28 mol/l, was added dropwise under vigorous stirring. The final mixture was stirred at room temperature for 24 h. Subsequently, TiO_2_-sol was spin cast on FTO conducting glass substrates at 3,000 rpm for 40 s. Then, the samples were annealed at 500°C for 30 min in a muffle furnace. The typical thickness of TiO_2_ is 25 nm. For the active layer, P3HT (used as received) was dissolved in 1,2-dichlorobenzene to produce an 18-mg/ml solution, followed by blending with PCBM (used as received) in 1:1 weight ratio [22]. The blend was stirred for 24 h in a nitrogen-filled glovebox before spin coating on top of the TiO_2_ film surface. Then, the samples were annealed at 150°C for 10 min on a hot plate in the glovebox. The typical film thickness of P3HT:PCBM was about 100 nm. Finally, 1 nm of MoO_3_, 10 nm of Ag, and *x* nm (*x* =20, 40, 60, and 80 nm) of MoO_3_ were thermally evaporated in sequence under high vacuum (5 × 10^-4^ Pa) without disrupting the vacuum. The deposition rate which was monitored with a quartz-oscillating thickness monitor (CRTM-9000, ULVAC, Methuen, MA, USA) was about 0.05 nm/s. The active area of the device was about 4 mm^2^.

Current density-voltage (*J-V*) characteristics were measured using a computer-programmed Keithley 2400 source meter (Keithley 2400, Keithley Instruments, Inc., Cleveland, OH, USA) under AM1.5G solar illumination using a Newport 94043A solar simulator (Newport 94043A, Oriel, Irvine, CA, USA). The intensity of the solar simulator was 100 mW/cm^2^. Light intensity was corrected by a standard silicon solar cell. The transmission and reflection spectra were measured using ultraviolet/visible (UV-VIS) spectrometer (Carry 5000, Agilent Technologies, Inc., Santa Clara, CA, USA). The resistivity and sheet resistance were measured using four-point probe resistivity measurement (JG SZT-C).

## Results and discussion

**Figure 2:**
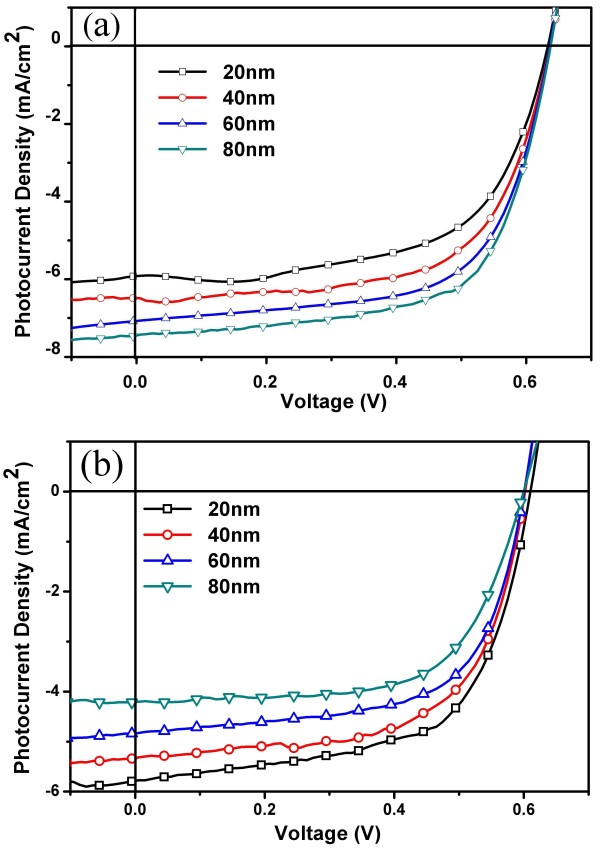
***J*-*V *characteristics.***J*-*V* characteristics of device FTO/nc-TiO_2_/P3HT:PCBM/MoO_3_ (1 nm)/Ag (10 nm)/MoO_3_ (*x* nm) (*x* =20, 40, 60, and 80 nm) depending on the thickness of the MoO_3_ capping layer when illuminated from **(a)** the ITO side and **(b)** the MoO_3_/Ag/WO_3_ side.

As shown in Figure 2a, the *J-V* characteristic curves of the device FTO/nc-TiO_2_/P3HT:PCBM/MoO_3_ (1 nm)/Ag (10 nm)/MoO_3_ (*x* nm) (*x* =20, 40, 60, and 80 nm) under AM1.5G solar illumination of 100 mW/cm^2^ in ambient air when illuminated from the FTO side (bottom). The detailed results are given in Table 1. Compared with our previous results [19] (the device has a structure of ITO/nc-TiO_2_/P3HT:PCBM/MoO_3_ (1 nm)/Ag (10 nm)/MoO_3_ (*x* nm)), there is a similar variation that the PCE increases with increasing MoO_3_ thickness when illuminated from the FTO electrode. It is known that reflectance of the top electrode plays an important role in trapping light for the active layer to reabsorb. The reflectance peaks of the MoO_3_/Ag/MoO_3_ electrode are redshifted and would match better to the absorption spectra of the active layer (400 to 650 nm) when the thickness of the MoO_3_ capping layer increases [19,20]. As a result, high PCE is achieved for FTO substrate inverted PSCs, which shows about 75% increases compared to the reported ITO substrate device at different thicknesses of the MoO_3_ capping layer of the MoO_3_/Ag/MoO_3_ transparent electrode films. The PCE increases from 1.40% to 2.43%, 1.55% to 2.62%, 1.64% to 2.87%, and 1.76% to 3.09% at MoO_3_ capping layer thicknesses of 20, 40, 60, and 80 nm, respectively.

**Table 1:** Characteristic data of semitransparent inverted polymer solar cells

**Device (nm)**	**Illumination**	***J***_**sc **_**(mA/cm**^**2**^**)**	***V***_**oc **_**(V)**	**FF (%)**	**PCE (%)**	***R***_**s **_**(Ω cm)**	***R***_**sh **_**(Ω cm)**
20	Bottom	6.00	0.64	63.28	2.43	14.01	713.78
20	Top	5.78	0.61	62.11	2.19	13.28	1,005.03
40	Bottom	6.48	0.64	63.18	2.62	12.53	1,247.99
40	Top	5.32	0.60	62.34	1.99	11.18	1,075.78
60	Bottom	7.07	0.64	63.43	2.87	10.61	629.88
60	Top	4.83	0.60	63.49	1.84	12.82	938.78
80	Bottom	7.45	0.64	64.81	3.09	10.72	898.55
80	Top	4.20	0.60	64.28	1.62	18.75	1,643.84

Different thicknesses of the MoO_3_ capping layer illuminated from the ITO (bottom) and MoO_3_/Ag/MoO_3_ (top) sides.

When illuminated from the MoO_3_/Ag/MoO_3_ electrode side (top), the efficiency decreases from 2.19% to 1.62% with increasing thickness of MoO_3_. This phenomenon, which is similar with that of the reported ITO substrate devices (0.96% to 0.60%), might result from the reduced transmittance of MoO_3_/Ag/MoO_3_ between 400 and 650 nm when increasing the MoO_3_ thickness. Figure 2b shows the *J*-*V* characteristic curves. The detailed results are given in Table 1. Meanwhile, PCE for FTO substrate inverted PSCs shows about 150% increase compared to the reported ITO substrate. The PCE increases from 0.96% to 2.19%, 0.82% to 1.99%, 0.72% to 1.84%, and 0.60% to 1.64% at MoO_3_ capping layer thicknesses of 20, 40, 60, and 80 nm, respectively.

One reason of these increases might be that the impact of FTO substrate samples on the environment is reduced, in which the process was in a nitrogen-filled glovebox while the reported process was in the air. The second reason might be that the resistivity of the ITO substrate increases after annealing treatment at high temperature while the resistivity of the FTO substrate does not. For ITO, the oxygen hole as carrier reduction causes the decrease of conductivity after annealing. The third reason might be that the light transmittance of the FTO substrate was a little higher than that of the ITO substrate.

**Figure 3:**
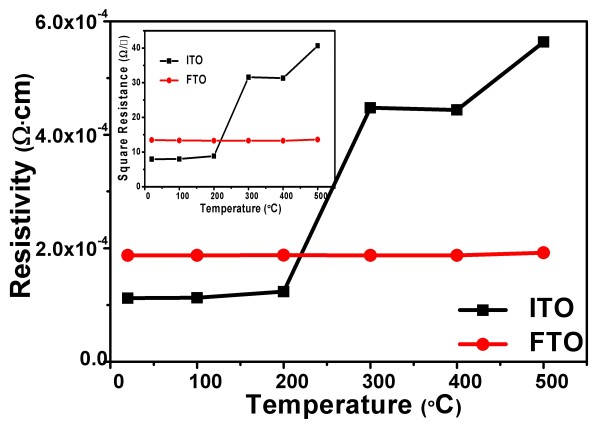
**Resistivity of ITO and FTO substrates after annealing treatment at different temperatures.***T* =20°C, 100°C, 200°C, 300°C, 400°C, and 500°C for 30 min. The inset shows the corresponding square resistance.

**Figure 4:**
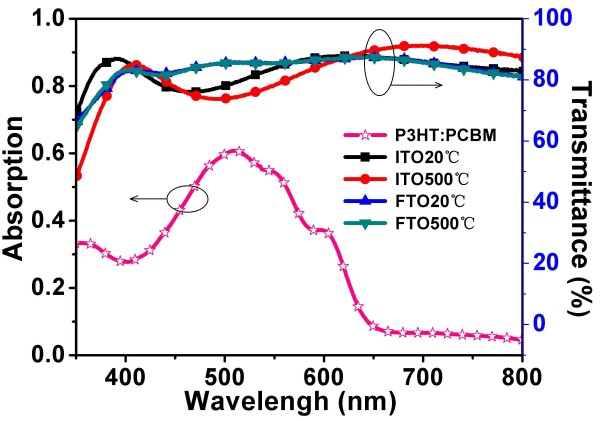
**Transmittance spectra of ITO and FTO substrates after annealing treatment at 20°C and 500°C.** The hollow star line is the absorption spectra of P3HT:PCBM active layer.

Here, we measured the resistivity of ITO and FTO substrates after annealing treatment at different temperatures (*T* =20°C, 100°C, 200°C, 300°C, 400°C, and 500°C) for 30 min. The curve is shown in Figure 3. For the ITO substrate, resistivity has a little change while annealing temperature is below 200°C and increases while annealing temperature is more than 300°C obviously. For the FTO substrate, resistivity has little change while annealing temperature is below 500°C. The inset in Figure 3 is the corresponding square resistance. For the ITO substrate, square resistance is 7.96 Ω/□ when annealing temperature is at 20°C and 40.65 Ω/□ at 500°C. It is about five times over. For the FTO substrate, square resistance is 13.48 Ω/□ when annealing temperature is at 20°C and 13.61 Ω/□ at 500°C which are of almost equal values. Because the oxygen holes as conductive carriers in the ITO decrease after 300°C annealing, the resistivity and sheet resistance increase. For FTO, this temperature is above 500°C. This is well matched with the causes.Figure 4 shows the transmittance spectra of ITO and FTO substrates after annealing treatment at 20°C and 500°C for 30 min and the absorption spectra of the P3HT:PCBM active layer. The absorption spectra range of the active layer is approximately 400 to 650 nm. In this range of wavelength, it can be seen that the transmittance of the FTO substrate is over 85% which is higher than that of the ITO substrate (between 75% and 85%). The active layer of the FTO substrate device can receive more sunlight than that of the ITO substrate device in a wavelength range of 400 to 650 nm. Meanwhile, the transmittance has been roughly unchanged after annealing at 500°C for the FTO substrate. For the ITO substrate, the transmittance has reduced and has a slight redshift. It might be caused by the reduction of oxygen vacancies.

## Conclusions

In summary, we demonstrated conductive FTO substrate semitransparent inverted PSCs with high-temperature annealing of nc-TiO_2_ as an electron-selective layer. The performances of PSCs with different substrates and different thicknesses of the MoO_3_ capping layer are investigated and compared. As a result, higher PCE was achieved for FTO substrate semitransparent inverted PSCs than that for the ITO substrate both illuminated from the bottom side and from the top side. For structurally identical PSCs, with increasing thickness of the MoO_3_ capping layer, the PCE is enhanced when illuminated from the bottom side but decreased when illuminated from the top side.

## Competing interests

The authors declare that they have no competing interests.

## Authors’ contributions

FL carried out the experiments, participated in the sequence alignment, and drafted the manuscript. CC participated in the device preparation. FT, CL, GY, LS, and WZ were involved in the UV-VIS and resistivity measurement analysis of devices. All authors read and approved the final manuscript.

## References

[B1] Sariciftci NS Smilowitz L Heeger AJ Wudl F Photoinduced electron transfer from a conducting polymer to buckminsterfullerene Science 1992 25 1474 1476 17755110 10.1126/science.258.5087.1474

[B2] Chen HY Hou JH Zhang SQ Liang YY Yang GW Yang Y Yu LP Wu Y Li G Polymer solar cells with enhanced open-circuit voltage and efficiency Nat Photonics 2009 3 649 653 10.1038/nphoton.2009.192

[B3] Lungenschmied C Dennler G Neugebauer H Sariciftci SN Glatthaar M Meyer T Meyer A Flexible, long-lived, large-area, organic solar cells Sol Energ Mat Sol C 2007 91 379 384 10.1016/j.solmat.2006.10.013

[B4] Krebs FC Fabrication and processing of polymer solar cells: a review of printing and coating techniques Sol Energ Mat Sol C 2009 93 394 412 10.1016/j.solmat.2008.10.004

[B5] Sun Y Takacs CJ Cowan SR Seo JH Gong X Roy A Heeger AJ Efficient, air-stable bulk heterojunction polymer solar cells using MoO_x_ as the anode interfacial layer Adv Mater 2011 23 2226 2230 10.1002/adma.201100038 21469222

[B6] Yang TT Wang M Duan CH Hu XW Huang L Peng JB Huang F Gong X Inverted polymer solar cells with 8.4% efficiency by conjugated polyelectrolyte Energ Environ Sci 2012 5 8208 8214 10.1039/c2ee22296e

[B7] He Z Zhong C Su S Xu M Wu H Cao Y Enhanced power-conversion efficiency in polymer solar cells using an inverted device structure Nat Photonics 2012 6 591 595

[B8] You JB Dou LT Yoshimura K Kato T Ohya K Moriarty T Emery K Chen CC Gao J Li G Yang Y A polymer tandem solar cell with 10.6% power conversion efficiency Nat Commun 2013 4 1446 23385590 10.1038/ncomms2411 PMC3660643

[B9] Deibel C Dyakonov V Polymer–fullerene bulk heterojunction solar cells Rep Prog Phys 2010 73 096401-1-39

[B10] Sista S Hong ZR Park M-H Xu Z Yang Y High-efficiency polymer tandem solar cells with three-terminal structure Adv Mater 2010 22 77 80 10.1002/adma.200901453 20217804

[B11] Kim JY Lee K Coates NE Moses D Nguyen TQ Dante M Heeger AJ Efficient tandem polymer solar cells fabricated by all-solution processing Science 2007 317 222 225 10.1126/science.1141711 17626879

[B12] Giles EE Victor MB Alain G Snaith HJ Neutral color semitransparent microstructured perovskite solar cells ACS Nano 2014 8 591 598 10.1021/nn4052309 24467381

[B13] Ameri T Dennler G Waldauf C Azimi H Seemann A Forberich K Hauch J Scharber M Hingerl K Brabec CJ Fabrication, optical modeling, and color characterization of semitransparent bulk heterojunction organic solar cells in an inverted structure Adv Funct Mater 2010 20 1592 1598 10.1002/adfm.201000176

[B14] Li G Chu CW Shrotriya V Huang J Yang Y Efficient inverted polymer solar cells Appl Phys Lett 2006 88 253503 10.1063/1.2212270

[B15] Shrotriya V Hsing-En E Li G Yao Y Yang Y Efficient light harvesting in multiple-device stacked structure for polymer solar cells Appl Phys Lett 2006 88 064104 10.1063/1.2172741

[B16] Hadipour A Boer B Wildeman J Kooistra FB Hummelen JC Turbiez M Wienk MM Janssen RAJ Blom PWM Solution-processed organic tandem solar cells Adv Funct Mater 2006 16 1897 1903 10.1002/adfm.200600138

[B17] Kim HP Lee HJ Yusoff ARM Jang J Semi-transparent organic inverted photovoltaic cells with solution processed top electrode Sol Energ Mat Sol C 2013 108 38 43

[B18] Yu WJ Shen L Meng FX Long YB Ruan SP Chen WY Effects of the optical microcavity on the performance of ITO-free polymer solar cells with WO3/Ag/WO3 transparent electrode Sol Energ Mat Sol C 2012 100 226 230

[B19] Tao C Xie GH Liu CX Zhang XD Dong W Meng FX Kong XZ Shen L Ruan SP Chen WY Semitransparent inverted polymer solar cells with MoO3/Ag/MoO3 as transparent electrode Appl Phys Lett 2009 95 053303 10.1063/1.3196763

[B20] Li FM Ruan SP Xu Y Meng FX Wang JL Chen WY Shen L Semitransparent inverted polymer solar cells using MoO_3_/Ag/WO_3_ as highly transparent anodes Sol Energ Mat Sol C 2011 95 877 880 10.1016/j.solmat.2010.11.009

[B21] Tao C Ruan SP Zhang XD Xie GH Shen L Kong XZ Dong W Liu CX Chen WY Performance improvement of inverted polymer solar cells with different top electrodes by introducing a MoO3 buffer layer Appl Phys Lett 2008 93 193307 10.1063/1.3026741

[B22] Shrotriya V Li G Yao Y Moriarty T Emery K Yang Y Accurate measurement and characterization of organic solar cells Adv Funct Mater 2006 16 2016 2023 10.1002/adfm.200600489

